# Effect of Meropenem, Sulbactam, and Colistin Combinations on Resistance Gene Expression in Multidrug-Resistant *A. baumannii* Clinical Isolates from Panama

**DOI:** 10.3390/antibiotics14100999

**Published:** 2025-10-07

**Authors:** José Emigdio Moreno, Jordi Querol-Audi, Ariel Magallón Tejada, Juan R. Medina-Sánchez, Armando Durant Archibold

**Affiliations:** 1 Doctorate in Biosciences and Biotechnology, Faculty of Science and Technology, Universidad Tecnológica de Panamá, Panama City 0819-07289, Panama; 2 Clinical Microbiology, Instituto Conmemorativo Gorgas de Estudios de la Salud, Panama City 0816-02593, Panama; 3 Department of Biochemistry and Nutrition, Faculty of Medicine, Universidad de Panamá, Panama City 0830-00929, Panama; 4 Sistema Nacional de Investigación (SNI), Secretaría Nacional de Ciencia y Tecnología (SENACYT), Panama City 0816-02852, Panama; 5 Biomedical Experimental Station, Instituto Conmemorativo Gorgas de Estudios de la Salud, Panama City 0816-02593, Panama; amagallon@gorgas.gob.pa

**Keywords:** *Acinetobacter baumannii*, antibiotic susceptibility, kill-curve assay, gene expression

## Abstract

Background: Given the increasing problem of antibiotic resistance in *A. baumannii*, this study examines in vitro how combinations of colistin, meropenem, and sulbactam influence the expression of genes associated with multiresistance in this pathogen. Methods: Three multidrug-resistant strains, isolated from clinical infections in Panama (2022–2023), were identified using Vitek 2 compact. Susceptibility by broth microdilution, qualitative synergy, time-kill curves, and gene expression analysis by quantitative PCR were performed. Results: Synergistic effects were observed for the colistin–meropenem combination in all three strains, while the sulbactam–colistin combination exhibit synergy only in one of the *A. baumannii* isolates. Time-kill assays revealed bactericidal effects for the colistin–meropenem and sulbactam–colistin combinations. qPCR analyses indicated that colistin, meropenem, and sulbactam modified the expression of the genes under study. Colistin–meropenem and meropenem–sulbactam combinations decreased the expression of *blaADC* and *blaOXA-51*, while sulbactam–colistin did not have a significant effect. *carO* expression levels were not reduced with any antibiotic combination, while *adeB* expression was reduced with all the combinations tested. *omp33–36* expression varied depending on the antibiotic and strain. Conclusions: Therefore, this study offers a new perspective on how rational combinations of clinically used antibiotics have the potential to modulate gene expression and contribute to the control of MDR strains, indicating that high-dose combination therapy with sulbactam and colistin could offer improved efficacy in treating multidrug resistant *Acinetobacter baumannii* infections.

## 1. Introduction

Antibiotics are essential agents in modern medical practice that have enabled safe surgeries, organ transplants, controlled therapies for chronic diseases, and their influence on lengthening human life expectancy, is a fact we take for granted [1]. Today, resistance mechanisms threaten their medical use, due to the emergence and spread of multidrug-resistant bacteria. *Acinetobacter baumannii* is the main pathogen associated with healthcare worldwide [2], presenting the greatest challenges in terms of treatment options, pharmacokinetics, bioavailability, and its ability to acquire mobile resistance elements [3,4], spread easily [5], and develop a wide range of carbapenem resistance mechanisms [6,7,8]. Combined with the limited number of in vitro studies using pathogenic strains, these factors highlight the need to understand how new treatment strategies using existing antibiotics can help suppress resistance in this bacterium.

Resistance mechanisms in *A. baumannii* affect a wide variety of antibiotics and allow sufficient plasticity to evade most effective treatments, concerning intensive care units and critically ill patients. Among these mechanisms, we find class C β-lactamases such as the constitutive Acinetobacter-derived cephalosporinases *blaADC*, carbapenemases including intrinsic class D carbapenemases such as *blaOXA-51* [3,4], porins involved in the exchange of basic amino acids such as ornithine (*carO*), cellular adherence and virulence (*omp33–36*), and active efflux systems such as Resistance Nodulation Division pumps of the *adeABC* type (where the *adeB* gene has a primary role as a channel protein) [5].

The use of monotherapy in multidrug-resistant bacteria is associated with unfavorable outcomes; if the patients are critically ill, refractoriness and the lack of therapeutic antibiotic options are the main cause of life-threatening complications [6]. Combination therapies are increasingly considered as strategies to enhance the effectiveness of the few available antibiotics; this approach is useful in bacteria such as *A. baumannii* [7]. Antibiotics such as colistin, which can cause nephrotoxicity, are the last-line clinical options for severe infections in soft tissues or the upper respiratory tract [8]; hence, research on optimized combinations of β-lactams and polymyxins, such as meropenem and colistin, respectively, should be prioritized. Recently, a particular focus has been placed on the inclusion of β-lactamase inhibitors, such as sulbactam, with the aim of developing more effective inhibitory strategies, especially against carbapenem-resistant *A. baumannii* (CRAB) [6,9].

Antibiotic resistance in *A. baumannii* represents a growing challenge to global public health, primarily due to the drastic limitation of therapeutic options for nosocomial infections. Considering this, the present study provides a comprehensive analysis of the phenotypic and genomic effects resulting from drug combinations applied to carbapenem-resistant *A. baumannii* strains.

In vitro susceptibility tests, qualitative synergy tests, kill curves, and gene expression analysis by qPCR were performed to determine how combinations of colistin, meropenem, and sulbactam alter the expression of different genes involved in multidrug resistance such as *blaADC*, *blaOXA-51*, *carO*, *omp33–36*, and *adeB* in *A. baumannii* clinical isolates. These findings will contribute to a better understanding of the complex interaction between combination therapies and the expression of resistance genes in *A. baumannii* in order to develop more effective therapeutic strategies.

## 2. Results

### 2.1. Antibiotic Susceptibility

**Figure 1:**
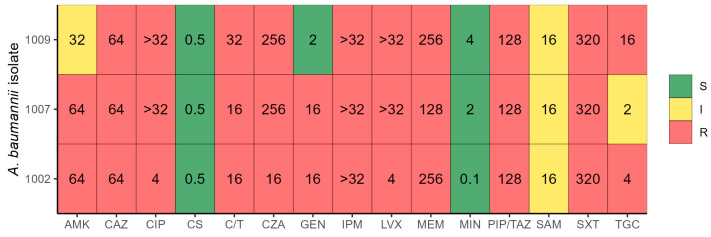
Antibiotic resistance profile of three *A. baumannii* isolates. Color indicates the resistance profile (S—sensitive, I—intermediate, R—resistant) and the value inside each box indicates the antibiotic MIC. AMK—amikacin, CAZ—ceftazidime, CIP—ciprofloxacin, CS—colistin, C/T—Ceftolozane/Tazobactam, CZA—Ceftazidime/Avibactam, GEN—gentamicin, IPM—imipenem, LVX—levofloxacin, MEM—meropenem, MIN—minocycline, PIP/TAZ—piperacillin–tazobactam, SAM—ampicillin–sulbactam, SXT—trimethoprim–sulfamethoxazole, TGC—tigecycline.

Biochemical identification using Gram-negative cards, along with the detection of the *blaOXA-51* gene, confirmed that all the isolates belonged to the *Acinetobacter baumannii* species. Regarding their phenotypic resistance profiles and based on the classification scheme proposed by Jiménez-Pearson et al. [10], all three isolates were categorized as multidrug-resistant (MDR), exhibiting resistance to multiple antibiotic classes. *A. baumannii* 1002 showed the highest level of resistance, being non-susceptible to 8 out of 11 antibiotics tested, followed by strains 1007 (7/11) and 1009 (6/11) (see Figure 1).

Notably, as no established clinical breakpoints exist for ceftazidime/avibactam and ceftolozane/tazobactam, the minimum inhibitory concentrations (MICs) for these drugs were evaluated using E-test strips. All three isolates exhibited the highest MIC values for these combinations in vitro.

The isolates were not susceptible to the following antibiotic classes: β-lactams, β-lactam/β-lactamase inhibitor combinations, cephalosporins across all generations, and carbapenems. Meropenem displayed MIC values greater than 128 µg/mL, and all three strains showed intermediate resistance to ampicillin–sulbactam. Colistin and minocycline remained active against all three isolates, with MICs below 0.5 µg/mL and 4 µg/mL, respectively. Tigecycline demonstrated activity only against strain 1007. Other affected antibiotic classes included fluoroquinolones, folate synthesis inhibitors, and aminoglycosides (see Table 1).

### 2.2. Detection of Resistance Genes

The three strains were negative for the most common carbapenem resistance genes such as *bla*_KPC_, *bla*_NDM-1_, *bla*_VIM_, *bla*_IMP_, and *bla*_OXA-48_. Additionally, they were positive for constitutive genes such as *blaOXA-51*, *blaADC*, *carO*, *adeB*, and *omp33–36* and belonged to the same clone ST79^Pas^/ST124^Oxf^.

### 2.3. Qualitative Synergies

The MIC values obtained for colistin (CS), meropenem (MEM), and sulbactam (SUL) were ≤0.5 µg/mL, ≥128 µg/mL, and ≤16 µg/mL, respectively. For the antibiotic mixtures, the MIC values of each and the calculated FICI can be seen in Table 2. For the three strains, the synergistic combinations were limited to colistin–meropenem (CM); only the *A. baumannii* 1007 strain showed synergy against the sulbactam–colistin (SC) mixture. For all the strains, the meropenem–sulbactam (MS) mixture had no effect at 24 h of incubation.

### 2.4. Time-Kill Kinetics Assays

**Figure 2:**
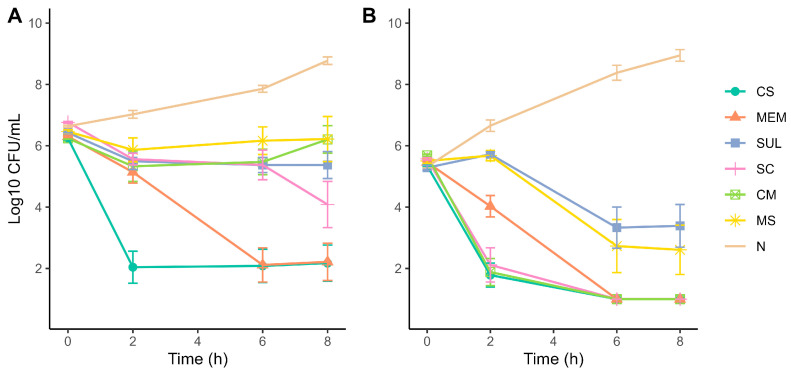
Time-kill curves of *A. baumannii* strains exposed to single and combined antibiotics at (**A**) 1xMIC and (**B**) 4xMIC. Antibiotics: CS—colistin, MEM—meropenem, SUL—sulbactam, SC—colistin + sulbactam, CM—colistin + meropenem, MS—meropenem + sulbactam, N—untreated control/no antibiotic. Experiments were performed in biological triplicates.

Time-kill curve assays were performed using colistin, meropenem, sulbactam and their combinations (colistin–meropenem [CM], meropenem–sulbactam [MS], and sulbactam–colistin [SC]) at 1xMIC and 4xMIC. Differences in growth inhibition were observed between the two MICs over time. At 1xMIC, colistin best bactericide effect, while the bactericidal effect of meropenem was not significantly different to colistin after 6 h. Antimicrobial combinations exhibit a slight delayed effect against bacterial growth; for example, SC effect was comparable to colistin and meropenem after 8 h and sulbactam drastically reduced the effect of meropenem when combined (Figure 2A). At 4xMIC, colistin and colistin combinations (CM and SC) exhibit bactericidal effect at 2 h, while bactericidal effect of all antibiotics and combinations shows no statistically significant difference after 6 h, except for sulbactam with lower bactericidal effect (Figure 2B). Furthermore, the bactericidal effect of meropenem was also reduced by sulbactam during the first hours of the assay at 4xMIC.

### 2.5. Gene Expression

For the gene expression assays, sub-MIC were used for all antibiotics, to ensure adequate logarithmic growth and good quality RNA. The average geometric basal expression of the *blaADC*, *blaOXA-51*, *carO*, *omp33–36*, and *adeB* genes was 25.49, 3.32, 5.04, 1.36, and 0.06 times, respectively. The increased expression of the *blaADC*, *blaOXA-51*, and *adeB* genes implies increased resistance to various antibiotics, particularly β -lactams

The effect on *blaADC* gene expression by colistin, meropenem, and sulbactam was 47.0, 36.14, and 44.94, respectively; the CM (4.98) and MS (5.17) combinations decreased it, and there was no effect for the SC mixture (20.01). Comparable results were obtained for *blaOXA-51* gene with expression values for the same antibiotics of 46.33, 15.21, and 38.82, respectively, while the CM and MS combinations decreased to values of 2.46 and 2.46, and SC treatment did not cause significant differences (11.29). In the case of *adeB*, in which the basal expression was low, the use of antibiotics alone such as meropenem (0.062) and sulbactam (0.566) slightly upregulated gene expression, while the CM (0.001), MS (0.001) and SC (0.014) mixtures reduced it even further. Colistin increased expression of this gene with an average value of 2.89.

**Figure 3:**
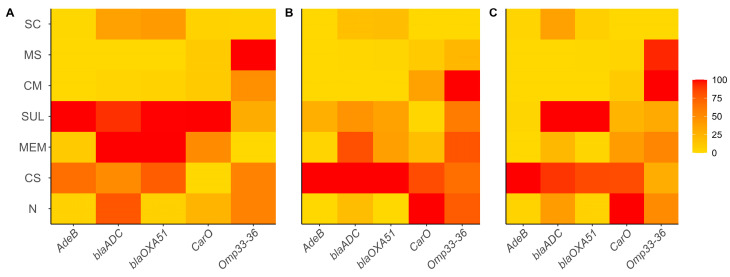
Relative expression of *adeB*, *blaADC*, *blaOXA-51*, *carO* and *omp33–36* genes from *A. baumannii* strains exposed to single and combined antibiotics. *A. baumannii* strains: (**A**) 1002, (**B**) 1007 and (**C**) 1009. Antibiotics: CS—colistin, MEM—meropenem, SUL—sulbactam, SC—colistin + sulbactam, CM—colistin + meropenem, MS—meropenem + sulbactam, N—untreated control/no antibiotic. Experiments were performed in biological duplicates.

Conversely, the underexpression of genes such as *carO* and *omp33* implies increased resistance to different antibiotics. For our assay, we found that *carO* expression was lower in colistin (3.0), meropenem (3.6), sulbactam (3.5), CM (2.0), MS (1.6), and SC (1.3) treatments. *omp33–36* showed the following expression values for colistin (1.16), meropenem (1.0), sulbactam (1.035), and the SC mixture (0.4), but also showed increases with the CM (1.7) and MS (1.6) mixtures (see Figure 3). By normalizing these values, gene expression varies from strain to strain and with each type of antibiotic to be tested; however, the expression of the *blaADC*, *blaOXA-51*, and *adeB* genes decreased due to the CM, MS, and SC treatments in most of the strains. In the case of *carO*, colistin and sulbactam had a decreasing effect, as well as CM and MS for *omp33–36* (see Figure 3).

## 3. Discussion

This study investigated the in vitro efficacy of various antibiotic combinations against MDR *A. baumannii* strains, isolated from severe human infections. We evaluated both the impact of these combinations on bacterial growth kinetics and on the expression of resistance-related genes. The main objective was to identify the most effective drug combinations to combat MDR *A. baumannii* infections.

All three strains exhibited similar resistance profiles and were classified as MDR, exhibiting susceptibility to only 3 of 11 reference antibiotics [10]. All strains were resistant to all tested β-lactams and displayed a high degree of resistance to β-lactam/β-lactamase inhibitor combinations such as ceftazidime/avibactam or ceftolozane/tazobactam, consistent with previous reports [11,12]. Regarding tigecycline susceptibility, one isolate was classified as resistant, one exhibited intermediate susceptibility, and one isolate was sensitive. Prior studies have linked such patterns to the presence of *ade*ABC-type RND pumps with variable susceptibility to tigecycline in *Acinetobacter* spp. [9,13]. Given its poor pharmacokinetics and observed indifference in checkerboard assays, tigecycline was excluded from subsequent synergy tests. Its limited penetration in blood and tissue models, from which the strains were originally isolated, further supports its unsuitability for synergy evaluations [14].

High MICs to meropenem (>128 µg/mL) are consistent with those observed for other MDR strains [8]. In the other hand, strains were susceptible to colistin; however, the clinical use of this antibiotic is complicated due to its nephrotoxicity, difficult dosage, thus limiting its application as monotherapy [15]. However, it was demonstrated as a promising candidate for testing combination therapy with meropenem and sulbactam, the latter of which acts as a β-lactamase inhibitor in *A. baumannii* [16,17].

Even though the most common carbapenemase genes (*blaKPC*, *blaNDM-1*, *blaVIM*, *blaIMP* and *blaOXA-48*) were not detected by PCR in this study, this is not surprising. While these enzymes are prevalent in *Enterobacterales*, class D carbapenemases along with overexpression of efflux pumps such as RND plays a more prominent role in β-lactam resistance in *Acinetobacter* spp. worldwide [18,19,20].

Qualitative checkerboard synergy assays showed that the most effective combinations were colistin–meropenem and sulbactam–colistin. These findings are consistent with previous in vitro and in vivo studies [9,17]. Although the checkerboard method has limited discriminatory power compared to time-kill assays [8], the colistin–meropenem combination achieved synergy in all strains and reduced bacterial counts by 3 Log_10_ CFU/mL at 24 h. However, despite its in vitro efficacy, its clinical application is compromised by the nephrotoxicity associated with colistin, and evidence from various studies indicates that it does not significantly reduce in vivo mortality [8,21,22]. Therefore, alternative combinations with other drugs would reduce toxicity, prevent hetero-resistance, and prolong the bactericidal effects [15,23,24].

Time-kill assays confirmed limited bactericidal activity of the meropenem–sulbactam combination at concentrations ≤ MIC of sulbactam but revealed a synergistic effect for the colistin–sulbactam combination that was not detected by checkerboard analysis [25]. Specifically, our data show that combinations of two (or more times) the MIC of sulbactam with MIC or sub-MIC levels of colistin have a synergistic bactericidal effect, by reducing 2 log_10_CFU/mL compared to colistin alone, consistent with what has been reported in in vitro or clinical trials [8,9,26]. Intermediate MICs for sulbactam indicated the presence of cephalosporinases such as *blaADC* or *blaTEM*-type beta-lactamases. Nevertheless, our results suggest the potential of sulbactam in combination with colistin for treating MDR or XDR *A. baumannii* [9] infections.

Our comprehensive analysis suggests that the combined regimen of sulbactam and colistin offers distinct advantages over colistin monotherapy in the management of *A. baumannii* infections. Specifically, prospective in vivo investigations have demonstrated that this combination yields a significantly superior early clinical response compared to colistin monotherapy (70% vs. 15.8%) [27]. Moreover, dose optimization studies indicate that higher sulbactam dosages, when combined with colistin, result in enhanced microbiological eradication, with a 12 g/day regimen achieving a 90.5% microbiological cure rate compared to 58.1% for 9 g/day [28]. Furthermore, the observed in vitro synergistic activity between sulbactam and colistin [29] suggests a potential mechanism to mitigate the emergence of colistin resistance, a critical concern with monotherapy. A pivotal pharmacokinetic advantage of this combined approach is sulbactam’s demonstrated adequate penetration into lung tissue [27], a characteristic essential for effective treatment of pulmonary infections where colistin penetration is often limited. Finally, analyses of adverse events indicate that the sulbactam–colistin combination maintains a renal safety profile, comparable to colistin monotherapy [30], and exhibits significantly less nephrotoxicity than colistin combined with other antibacterial agents [9].

This is the first study to correlate the effect of different antibiotic combinations with gene expression of β-lactamases (*blaADC* and *blaOXA-51*), porin membrane proteins, (*omp33–36* and *carO*) and the channel component *adeB* of the RND pump system *ade*ABC of *A. baumannii*. Both *blaADC* and *blaOXA-51* have been associated with increased resistance to beta-lactams such as cephalosporins [31] and carbapenems like meropenem [32], with or without the presence of mobile elements such as *IS*Aba. This study determined that, in addition to meropenem, colistin and sulbactam significantly increased the basal expression of *blaADC* compared to untreated controls, similarly to previous findings [33], suggesting complex gene regulation systems in this bacteria.

The upregulation of the *blaADC*, *blaOXA-51*, and *adeB* genes indicates increased resistance to multiple antibiotics, particularly β-lactams. Expression levels of *blaADC* (25.49) and *blaOXA-51* (3.32) rose to 47.0 and 44.94 following treatment with colistin, and to 46.33 and 38.82 after treatment with sulbactam, respectively.

These data support our hypothesis that in MDR strains, the overexpression might be associated with *IS*Aba-type insertion elements and that conventional treatments may inadvertently enhance resistance, which would partly explain the refractory response seen in vivo. Importantly, the combinations of CM and MS significantly reduced expression of these genes (ANOVA, *p* < 0.05), consistent with previous results [34]. Thus, we provide evidence that these combinations can influence gene regulation and bacterial survival.

In contrast, expression of the porin gene *carO*, a key channel for carbapenem uptake and, therefore, associated with increased resistance when downregulated, was modestly decreased by colistin and not significantly altered by other treatments. These findings suggest that *carO* is not a viable therapeutic target in our strains, which raises the question of whether other regulatory mechanisms may restore its expression.

Overexpression of the *ade*ABC genes is a response to a cellular accumulation of toxic substances due to an increase in membrane permeability [35] while their inhibition or reduction can partially reverse resistance. Unexpectedly, colistin increased the expression of *adeB*, a component of the *ade*ABC efflux system, which has been closely linked to multidrug resistance phenotypes [36]. Sulbactam also increased *adeB* expression but to a lesser extent. Notably, all three combinations (CM, SC, MS) significantly reduced *adeB* expression below baseline, providing novel insights into how these combinations may be useful in managing efflux pump-mediated resistance in *A. baumannii*. Finally, expression of *omp33–36*, a porin involved in adhesion, invasion, fibronectin binding, and carbapenem resistance [37], was not significantly affected by the CM and MS combinations, whereas the SC combination led to a twofold reduction in its expression. This downregulation may confer transient low virulence in the host, reinforcing the potential clinical relevance of these combinations [38], and the downregulation of *omp33–36* may also increase resistance to imipenem as previously reported [39].

## 4. Materials and Methods

### 4.1. Bacterial Identification and Susceptibility

The three strains obtained from clinical infections in Panama between the years 2022 and 2023 for infections conditions, such as bacteremia, pneumonia, and wounds, were selected based on their antimicrobial resistance profile, virulence factors, and genomic homology. Subsequently, the strains were cultured on MacConkey agar (Beckton Dickinson^®^, Franklin Lakes, NJ, USA) for 24 h at 35 °C, and then subcultured on trypticase soy agar (Beckton Dickinson^®^, Franklin Lakes, NJ, USA) for 24 h at 35 °C. Their biochemical identification was performed using GN cards and antimicrobial susceptibility tests with AST-N401 and AST-N403 cards, using the automated Vitek 2 Compact system (BioMerieux^®^, Lion, France). Additionally, the activity of meropenem, imipenem, ceftazidime–avibactam, ceftolozane–tazobactam, colistin, tigecycline, ciprofloxacin, and trimethoprim–sulfamethoxazole was evaluated using the epsilometric E-test^®^ method (BioMerieux^®^, France) on Müeller-Hinton II agar (Beckton Dickinson^®^, USA), following the manufacturer’s instructions. The determination of the minimum inhibitory concentration was performed by broth microdilution, following the guidelines provided in the M100-Ed34 document and the CLSI regulations. To ensure accuracy and reproducibility, the MIC assay was performed in biological triplicate for each antibiotic [40]. The antibiotics used in the study included meropenem, colistin, and sulbactam, all purchased in powder form (Sigma-Aldrich©, St. Louis, MO, USA). The procedure was conducted in sterile 96-well plates with a concave bottom, using cation-adjusted Müeller-Hinton II broth (Beckton-Dickinson^®^, Franklin Lakes, NJ, USA). Incubation took place at 35 °C in aerobic conditions for 20–24 h.

### 4.2. Qualitative Synergy by Checkerboard

The experimental protocol adopted is based on the recommendations of Bellio et al. [41], which outlines a systematic method for evaluating combinations of antibiotics in 96-well plates. For this purpose, the concentrations of the antibiotics were set at four and two times the minimum inhibitory concentration (MIC), with the aim of performing cross-dilutions between the vertical and horizontal rows of the plate, which allowed generating 77 different combinations of two drugs. The determination of individual MICs was performed using the data obtained from the first column (1) and the last row (H), while the calculation of the fractional inhibitory concentrations (FIC) and the fractional inhibitory concentration index (FICI) facilitated the evaluation of pharmacological interactions. These interactions were classified as synergistic (<0.5), indifferent (0.5–4), and antagonistic (>4.0), according to the results [8]. The experiments involved the mixtures of colistin–meropenem, meropenem–sulbactam, and sulbactam–colistin, and were performed in biological duplicate to strengthen the reliability of the data. To evaluate cell viability, 0.015% sodium resazurin was added in a volume of 30 μL and incubated for 2 h at 35 °C. Color change from blue to pink indicated cell viability, while no color change indicated complete growth inhibition [42].

### 4.3. Detection of Antimicrobial Resistance Genes

A bacterial suspension adjusted to 0.5 McFarland (1.5 × 10^8^ CFU/mL) was prepared from a 24 h pure culture in trypticase soy broth (Beckton Dickinson^®^). After centrifugation at 8000× *g* for 10 min in molecular grade water (Promega^®^, Madison, WI, USA), pellet was subjected to DNA extraction using the DNeasy Blood & Tissue Kit (Qiagen^®^, Hilden, Germany), following manufacturer’s instructions. Detection of carbapenemase genes *bla*KPC, *bla*NDM-1, *bla*VIM, *bla*IMP, and *bla*OXA-48 was carried out by PCR using the primers specified in Table 3. Amplification was performed with the Master Mix 2X (Promega^®^) in a T100^®^ thermocycler (Bio-Rad^®^, Hercules, CA, USA). Electrophoresis was performed on 1.5% Qiagen agarose gels stained with Gel-Red™ (Olerup SSP^®^, Stockholm, Sweden), using a CompactM Biometra^®^ chamber run at 110 V for 45 min. Finally, results were recorded in a UVP Gelstudio^®^ transilluminator (Analytik Jena^®^, Jena, Germany).

### 4.4. Time-Kill Curve Assays

Following Krohn’s protocol [43], 100 μL of a bacterial suspension adjusted to 1.0 McFarland, from cultures in logarithmic phase were inoculated in 10 mL of Müller-Hinton II broth. Each suspension contained the following antibiotics at concentrations of 1xMIC and 4xMIC: 0.5 µg/mL colistin (C), 128 µg/mL meropenem (M), 16 µg/mL sulbactam (S), 0.5/128 µg/mL colistin/meropenem (CM), 128/16 µg/mL meropenem/sulbactam (MS), and 16/0.5 µg/mL sulbactam/colistin (SC) (1 × MIC assays); and 2 µg/mL, 512 µg/mL, 64 µg/mL, 2/512 µg/mL, 512/64 µg/mL, and 64/2 µg/mL, respectively (4 × MIC assays). A tube without antibiotics was included as control.

Time-kill assays were conducted in biological triplicates for each strain. Ten-fold serial dilutions in 1 mL of 0.85% NaCl were applied from 10^−1^ to 10^−7^. An amount of 10 µL of each dilution was inoculated in Müeller-Hinton II agar (Beckton-Dickinson^®^). This procedure was performed at intervals of 0, 2, 4, 6, 8, and 24 h. After incubation at 35 °C for 24 h, colonies were counted to calculate the CFU/mL using the following formula: CFU/mL = n(1/d)(100) where “n” is the colony number, “d” the dilution factor, and 100 the constant corresponding to the inoculum of 10 µL.

Differences between antibiotic treatments by time were analyzed using ANOVA with a significance level of 0.05, followed by Tukey’s HSD test to determine differences between groups. Analysis was performed using R v. 4.4.1.

### 4.5. RNA Extraction and Reverse Transcription

Each strain was inoculated in 5 mL of Müeller-Hinton II broth (Beckton-Dickinson^®^) along with a control without antibiotics. A total of 7 tubes containing sub-MIC of colistin, meropenem, sulbactam, and their mixtures (CM, MS, and SC) were used. After 22 h at 35 °C with shaking, cells were recovered and placed on ice and total RNA was extracted using the Norgen Biotek Corp© (Thorold, ON, Canada) column kit following the manufacturer’s protocol. All RNAs were kept at −80 °C until use. RNA concentration was measured in NanoDrop© equipment (Thermo Fisher Scientific^®^, Waltham, MA, USA). Reverse transcription was performed using a Quantitec^®^ Reverse Transcription kit (Qiagen© Germany) following manufacturer’s recommendations. cDNA products were quantified, and concentration was adjusted to approximately 65 ng/µL for qPCR assays.

### 4.6. qPCR Expression Assays

qPCR was performed to determine the expression of β-lactamase genes *blaADC* and *blaOXA-51*, porins *carO* and *omp33–36*, and the *adeABC* system channel gene, *adeB* (see Table 3). These genes were selected given their constitutive basal expression, contribution to multiresistance in *A. baumannii*, the possibility of use as a therapeutic target, and versatility of interacting with various antibiotics. The SYBR ™ Green PCR Master Mix (Thermo Fisher Scientific^®^) and the Applied Biosystem 7500 fast equipment (Thermo Fisher Scientific^®^) were used. Assays were performed in duplicate for each strain using sub-MIC concentration of antibiotic and the same mixtures used in the time-kill assay. For genes *blaADC* and *blaOXA-51*, the selected housekeeping references were as follows: *blaADC* was normalized using *16S1*; *carO* with cpn60; *omp33–36* with *rpoB*; and *adeB* was normalized using *16S2*, all in biological duplicates.

Gene expression analysis was performed using the 2^−ΔΔCt^ as described [44], using the *A. baumannii* ATCC19606 strain as control. Untreated strains were used to quantify basal expression. Changes in gene expression greater than 2 were considered as overexpression while changes less than 0.5 were interpreted as underexpression [32,44]. Results were analyzed using the GraphPad Prism 8 software package. Statistical significance was determined by two-way ANOVA, with Dunnett’s correction at a 95% confidence level. For normalization, 0% was assigned to the lowest value and 100% for the highest. Results are expressed as percentages, and Y tends toward 100% across all data points.

**Table 3:** Primers used for conventional PCR and qPCR amplification.

Gene	5′–3′	3′–5′	Source
Primers for conventional PCR			
*blaVIM*	AGTGGTGAGTATCCGACA	ATGAAAGTGCGTGGAGAC	[45]
*blaIMP*	GGYGTTTWTGTTCATACWTCKTTYGA	GGYARCCAAACCACTASGTTATCT	
*blaNDM-1*	AGCACACTTCCTATCTCGAC	GGCGTAGTGCTCAGTGTC	[46]
*blaKPC*	AACAAGGAATATCGTTGATG	AGATGATTTTCAGAGCCTTA	
*blaOXA48*	ATGCGTGTATTAGCCTTACGG	TGAGCACTTCTTTTGTGAATG	[47]
Primers for qPCR			
*blaADC*	TTATGCGGGCAATACACCA	CTGACAGAACCTAGCTCAAAAATG	[48]
*blaOXA-51*	CTATGGTAATGATCTTGCTCGTG	TGGTGGTTGCCTTATGGTG	
*16S1*	ACGGTCGCAAGACTAAAACTCA	GTATGTCAAGGCCAGGTAAGGT	
*carO*	AGCTTTACTTGCTGCTGGTG	CGAGCGCCTACTGGAATTA	[49]
cpn60	TTGACCGTGGTTATATCTCTCC	CGGATTTTCAAGTTCAGCAG	
*omp33–36*	GCTTATCAATTTGAAGTTCAAGGTC	GCTTGGTTTAAGAAAGCTGC	[37]
rpoB	TCCGCACGTAAAGTAGGAAC	ATGCCGCCTGAAAAAGTAAC	
*adeB*	GCAGAGCGTACTCGGAATGT	CCACTGAAACCCCATCCCAA	[50]
*16S2*	AGCTAACGCGATAAGTAGACCG	TGTCAAGGCCAGGTAAGGTTC	

ADC, *Acinetobacter*-derived cephalosporinase; *adeB*, component of the *ade*ABC efflux pump system; *carO*, carbapenem-associated resistance outer membrane protein; *cpn*60, bacterial chaperonin protein; *blaIMP*, imipenemase metallo-beta-lactamase; *blaKPC*, *Klebsiella pneumoniae* carbapenemase; *blaNDM-1*, New Delhi metallo-beta-lactamase; *omp33–36*, outer membrane protein of 33–36 kDa; *blaOXA-48*, oxacillinase-48 carbapenemase; *rpo*B, RNA polymerase beta subunit; *blaVIM*, Verona integron-encoded metallo-beta-lactamase; *16S1* rRNA, *16S2* ribosomal RNA.

## 5. Conclusions

Our findings are part of a new vision of how gene regulation mediated by the coherent mixture of antibiotics for clinical use can help control MDR and XDR strains of *A. baumannii*, supported by in vitro assays that demonstrated not only susceptibility to the antibiotic combinations tested but also their effects on bacterial growth kinetics. However, these findings are limited to clones present in strains from Panama and, therefore, similar research should be conducted in other Latin-American countries to assess their applicability to other clones in the region [51].

## Figures and Tables

**Table 1:** Clinical and epidemiological data and antimicrobial susceptibility profile of *A. baumannii* clinical isolates included in the study.

Isolate	State	Year	Clinical Sample	MIC µg/mL														
				SAM	PIP/TAZ	CAZ	IPM	MEM	CS	GEN	AMK	MIN	TGC	CIP	LVX	SXT	CZA	C/T
*A. baumannii* 1002	Panamá City	2022	Tracheal Secretion	16	128	64	>32	256	0.5	16	64	0.1	4	4	4	320	16	16
*A. baumannii* 1007	Panamá City	2022	Blood	16	128	64	>32	128	0.5	16	64	2	2	>32	>32	320	256	16
*A. baumannii* 1009	Panamá City	2023	Wound	16	128	64	>32	256	0.5	2	32	4	16	>32	>32	320	256	32

AMK—amikacin, CAZ—ceftazidime, CIP—ciprofloxacin, CS—colistin, C/T—ceftolozane/tazobactam; CZA—ceftazidime/avibactam, GEN—gentamicin, IPM—imipenem, LVX—levofloxacin, MEM—meropenem, MIN—minocycline, PIP/TAZ—piperacillin–tazobactam, SAM—ampicillin-sulbactam, SXT—trimethoprim–sulfamethoxazole, TGC—tigecycline. In vitro susceptibility according to CLSI M100-ed34. Clinical breakpoints for tigecycline based on Pearson et al. [10] MIC experiments were performed in biological triplicates.

**Table 2:** MIC and FICI analysis of colistin, meropenem, and sulbactam combinations against carbapenem-resistant *A. baumannii*. assays were performed in biological triplicates.

	MIC			FICI		
Strains	CS	MEM	SUL	CM	SC	MS
*A. baumannii* ATCC 19606	<0.25	<1	0.5			
*A. baumannii* 1002	0.5	128	16	0.5 (S)	0.62 (I)	0.56 (I)
*A. baumannii* 1007	0.5	128	16	0.3 (S)	0.5 (S)	1.5 (I)
*A. baumannii* 1009	0.5	256	16	0.3 (S)	1 (I)	0.75 (I)

CLSI breakpoints were used for interpretation of colistin and meropenem resistance. FICI (Fractional Inhibitory Concentration Index): S, synergy; I, indifferent.

## Data Availability

All the data that supports the findings of this study are available from the corresponding author JEM, upon reasonable request.

## Abbreviations

The following abbreviations are used in this manuscript:

MIC	Minimal inhibitory concentration
PCR	Polymerase chain reaction
GN	Gram-negative
XDR	Extreme drug resistance
MDR	Multidrug resistance
